# The Matron's Winter Holiday

**Published:** 1919-12-27

**Authors:** 


### THE MATRONS' AND SISTERS' DEPARTMENT.

### THE MATRON'S WINTER HOLIDAY.

RIGHT through the country Christmas brings to those in authority an immense access of work, not to say worry. Busy people on whom the well-being of many sick and helpless persons depends may be pardoned if they sometimes groan when their carefully-though-out routine is invaded and things undreamed of in normal times are expected to take place in their domain. At best Christmas-time brings a joyous inrush of outside help, a host of problems to deal with, a lengthening of hours far into the night, and a general quickening of resource and powers of triumphing over obstacles. At worst it exhausts the entire nursing staff and leaves them a prey to what may be called "Christmas nerves." But, for better or worse, the season is a landmark, and brings with it exceptional fatigues. Ought it not to be followed by a holiday for the matron, and for other heads of departments?

The winter holiday is a feature in most hospitals overseas. It is recognised over there that eleven months are too long for a woman on whom such diverse claims are continuously made to keep going without a breathing space. And in some hospitals in England the matron can command an occasional week-end, which may stretch perhaps to a parson's week. But these are exceptions. The woman who teaches enjoys the privilege of three breaks in her year's routine. The woman who nurses must be content with one, however high her position.

The proportion of matrons who break down and ultimately retire for reasons of health is notoriously large. It has little to do with hard work, which is one of the healthiest conditions man can enjoy. It has much to do with the worry which ensues when, after extra fatigue, the natural dictate of Nature to rest awhile has perforce to be disobeyed. Christmas festivities, prize distributions, the organisation of the next year's work, frequently combine to reduce the matron to sleeplessness or an unaccountable fit of neuralgia, or it may be only an uncommonly heavy cold, all symptoms of having "run down," and the year starts all wrong with her in consequence. Were she buoyed up through it all with the knowledge that her half-yearly rest were at hand, all would go better. The machine cheerfully works at high pressure for a time, but it needs overhauling and re-oiling afterwards.

It does not appear to be the business of anyone in particular to look after the matron in the hospital scheme of things. She feels herself responsible for the welfare of her household, and knows there is something wrong if the sick-rate in the institution staff goes up from one year to another. But how few Committees realise the need of the matron for special consideration until she succumbs at length to illness and they have to find a substitute. Everyone knows that a stitch in time will save nine so far as probationers are concerned. Who thinks of applying this dictum to the matron?

The notion of taking holiday twice a year instead of once is horrifying to some individuals in the nursing world, and not least to some matrons themselves. In the ballot arranged for the M.A.B. nurses whether or no the time off duty should be so arranged as to give them a half-yearly holiday, the voting went against the suggestion, though it is true a good many voted in favour of this double rest. The truth is that disturbance of routine is not favoured in hospitals. It is thought to make extra work, and the amount of rearrangement required is greatly exaggerated in anticipation. Like other reforms, the double holiday needs clever management if it is not to prove a burden. But in the hands of a capable administrator its very drawbacks work for good. The natural disinclination, felt by none more strongly than by matrons, to leave their responsibilities in other hands is a defect. Every chief ought to have a capable understudy who knows all the secrets of the department and can be depended on to keep things straight if the head fails. Now, there is no way of providing a good understudy comparable to that of leaving her in charge of the department periodically. Her defects then come into the open, and can be remedied by candid criticism. Confidence in her powers grows with practice, and the hospital is the richer in the possession of an official ready to take the reins in an emergency.

If to the winter holiday the excellent overseas plan were adopted of giving the matron six months' leave on full pay after seven years' service, we should hear less of irreplaceable matrons breaking down in health before their work be half accomplished.

